# MicroRNAs as Molecular Targets for Cancer Therapy: On the Modulation of MicroRNA Expression

**DOI:** 10.3390/ph6101195

**Published:** 2013-09-30

**Authors:** Pedro M. Costa, Maria C. Pedroso de Lima

**Affiliations:** 1 CNC-Center for Neuroscience and Cell Biology, University of Coimbra, 3004-517 Coimbra, Portugal; E-Mail: pedrocosta24@gmail.com; 2 Department of Life Sciences, Faculty of Science and Technology, University of Coimbra, 3001-401 Coimbra, Portugal

**Keywords:** microRNA (miRNA), anti-miRNA oligonucleotides, miRNA mimics, cancer therapy, viral and non-viral carriers

## Abstract

The discovery of small RNA molecules with the capacity to regulate messenger RNA (mRNA) stability and translation (and consequently protein synthesis) has revealed an additional level of post-transcriptional gene control. MicroRNAs (miRNAs), an evolutionarily conserved class of small noncoding RNAs that regulate gene expression post-transcriptionally by base pairing to complementary sequences in the 3' untranslated regions of target mRNAs, are part of this modulatory RNA network playing a pivotal role in cell fate. Functional studies indicate that miRNAs are involved in the regulation of almost every biological pathway, while changes in miRNA expression are associated with several human pathologies, including cancer. By targeting oncogenes and tumor suppressors, miRNAs have the ability to modulate key cellular processes that define the cell phenotype, making them highly promising therapeutic targets. Over the last few years, miRNA-based anti-cancer therapeutic approaches have been exploited, either alone or in combination with standard targeted therapies, aiming at enhancing tumor cell killing and, ideally, promoting tumor regression and disease remission. Here we provide an overview on the involvement of miRNAs in cancer pathology, emphasizing the mechanisms of miRNA regulation. Strategies for modulating miRNA expression are presented and illustrated with representative examples of their application in a therapeutic context.

## 1. Introduction

Over the last decade, miRNAs have emerged as important players in the highly complex world of gene regulation. A large number of studies involving transcriptomic, proteomic and bioinformatic approaches indicate that these small RNA molecules can regulate over 30% of all protein-coding genes and play a pivotal role in the most basic cellular processes—such as embryonic development, cell differentiation, metabolism, proliferation and cell death—in a wide range of invertebrate and vertebrate organisms, including humans [1,2,3,4]. Widespread influence of miRNAs is also observed in different physiological responses, like cardiovascular development [5], stem cell differentiation [6], immune response [7], insulin secretion [8] and anti-viral defense [9]. Since the miRNA milieu has a broad effect over diverse genetic and molecular pathways, it is not surprising that abnormal miRNA expression has been associated with several human diseases, including cardiovascular and neurological disorders [10,11], diabetes [12] and cancer [13]. In this review, we briefly describe the miRNA biogenesis and miRNA-mediated gene silencing mechanisms and discuss the current knowledge on how miRNAs can act as oncogenes and tumor suppressors. We also address recent advances on the application of miRNA-based therapeutic approaches to cancer.

## 2. MiRNA Biogenesis and Gene Silencing Mechanisms

The synthesis of miRNAs from their initial chromosome encryption to the final mature form is a highly regulated stepwise process that takes place in the cell nucleus and cytoplasm, and may interact with other important cellular functions, including splicing. In the biologically predominant canonical pathway, miRNAs are processed from 5'-capped and 3'-polyadenylated precursor molecules, designated primary miRNAs (pri-miRNAs), which are subsequently cleaved by a microprocessor complex that includes the RNase III enzyme Drosha and the double stranded (ds) RNA-binding protein DGCR8, to produce a 70-nucleotide (nt) hairpin-structured miRNA precursor (pre-miRNA). The pre-miRNA is exported from the nucleus to the cytoplasm via the transporter exportin-5, in a GTP-dependent process, where it is cleaved by a multiprotein complex that includes the endonuclease Dicer and the RNA-binding protein TAR (TRBP), yielding a miRNA duplex of approximately 21 to 23 nucleotides, with 2-nt overhangs at the 3' ends. In addition to the biologically prevalent canonical pathway, where miRNA precursor molecules are produced by the action of the microprocessor complex, an alternative (non-conventional) pathway that generates miRNA precursors by splicing-mediated cleavage of short-hairpin introns (mirtrons) was discovered and characterized in invertebrates [14].

Following the processing of the hairpin-containing pre-miRNA into a linear dsRNA molecule, effector miRNA-containing ribonucleoprotein complexes (miRNPs) are assembled through a dynamic (and as yet not fully understood) process, that involves recruitment of one of the four argonaute (Ago) proteins [15], a glycine-tryptophan repeat-containing protein of 182 kDa (TNRC6, also known as GW182), as well as several other proteins (such as the CCR4-NOT deadenylase complex), which probably function as miRNP assembly or regulatory factors [16,17]. The functional role of the miRNP complex in the miRNA-guided RNA silencing pathway is to recognize the miRNA guide in the miRNA-miRNA* duplex, pair it with its target mRNA and prevent mRNA translation [17].

With very few exceptions, animal miRNAs regulate gene expression by imperfect base pairing with sequences on the 3'-UTR of target mRNAs and further miRNP-mediated inhibition of translation and/or mRNA destabilization [2,18]. Functional studies, as well as computational approaches, have shown that perfect or near-perfect complementarity between the mRNA and the nucleotides 2–8 on the 5'-region of the miRNA, also known as the “seed” region, is determinant for target mRNA recognition by the miRNA [19,20]. Insufficient 5'-pairing can nevertheless be partially compensated by strong base-pairing between the 3'-region of the miRNA and the target mRNA [19,21]. While the “seed” sequence of the mature miRNA dictates which mRNAs it potentially interacts with, it is the protein components of the miRNP complex (especially GW182) that direct and execute the silencing of target mRNAs [22].

The current knowledge indicates that miRNAs repress the initiation of translation by interfering with key components of the initiation step machinery, namely with 5' cap recognition and 40S small ribosomal subunit recruitment, thus hampering the association between the 60S ribosomal subunit and the 40S initiation complex, and thereby preventing the formation of an active ribosomal complex [23] or binding to PABP, the poly(A)-binding protein attached to the 3' end of the mRNA that is involved in the circularization of the mRNA [24]. In addition to the repression of translation initiation, miRNAs can repress mRNA translation at the post-initiation steps by inhibiting ribosome elongation. Over the last few years, several studies surveying a large number of miRNA targets strongly indicate that mRNA destabilization (and consequent mRNA degradation) might nevertheless be a key factor in the decrease of protein levels caused by miRNAs. Unanimously, these studies found that miRNA repression results in concomitant changes in mRNA and protein levels, with changes in mRNA levels accounting for the majority, but not all, of the changes in protein abundance [25,26,27]. MiRNA-mediated mRNA decay is executed by the miRNP complexes through the recruitment of decay machinery components, leading to mRNA deadenylation and 5'-terminal decapping; the mRNA is subsequently degraded by the Xrn1 5'–3' exonuclease [28,29,30].

## 3. Role of MiRNAs in Cancer

The first indication that miRNA dysregulation could play a role in cancer was provided by Calin and colleagues, who demonstrated that two clustered miRNA genes, miR-15a and miR-16-1, were located in a region of the 13q14 locus that is commonly deleted in patients diagnosed with B-cell chronic lymphocytic leukemia (CLL) [31]. Soon after, Croce and colleagues showed that approximately 50% of annotated human miRNAs are located in cancer-associated genomic regions (CAGRs), including fragile sites, minimal regions of loss of heterozygosity, minimal regions of amplification or common breakpoint regions [32]. More recently, Kumar and colleagues demonstrated that global repression of miRNA maturation, through short hairpin (sh)RNA-mediated inhibition of several components of the miRNA processing machinery, promotes cellular transformation and tumorigenesis [33], thus supporting the idea that miRNAs play a crucial role in cancer progression. Indeed, several genome-wide miRNA-profiling studies provided evidence that distinct miRNA expression profiles distinguish tumors from normal tissues [34,35]. Different miRNA signatures were also associated with poor patient prognosis in lung cancer and CLL [36,37], indicating that miRNAs have potential to be used as diagnostic and prognostic markers.

Various genomic abnormalities were found to influence the activity of miRNAs, including deletions, amplifications or mutations involving miRNA loci, epigenetic silencing or dysregulation of transcription factors that target specific miRNAs. Loss or gain of miRNA function and its role in the development of cancer will be addressed below.

### 3.1. MiRNAs as Tumor Suppressors

**Figure 1:**
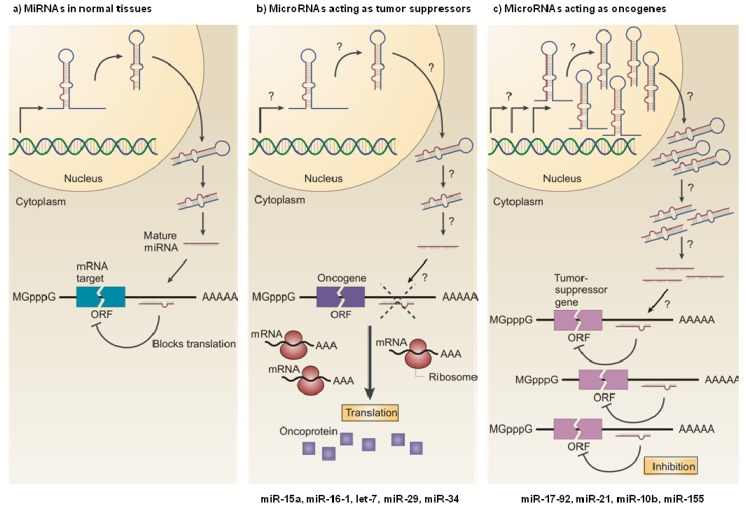
MicroRNAs as tumor suppressors and oncogenes. (**a**) In normal cells, miRNA transcription, processing and binding to complementary sequences in the target mRNA lead to the repression of their target genes, by either mRNA translation inhibition or mRNA degradation. (**b**) The reduced expression of a miRNA that acts as a tumor suppressor, as a result of chromosomal deletion or defects at any stage of miRNA biogenesis (indicated by question marks) leads to the increased synthesis of the miRNA-target oncoprotein (purple squares), and ultimately to the development of an oncogenic phenotype. (**c**) The increased expression of a miRNA that acts as an oncogene, as a result of (among others) amplification of the miRNA gene or constitutive promoter activation (indicated by question marks), leads to the repression of a miRNA-target tumor-suppressor gene (pink), which favors the development of an oncogenic phenotype. ORF: open reading frame; mGpppG: 7-methylguanosine. Reproduced with permission from [38].

MiRNAs can act as tumor suppressors when their reduced expression or loss of function contributes to the development of a malignant cell phenotype [3] (Figure 1). In this regard, accumulated data suggests that the cellular miRNA milieu is mostly composed of tumor suppressor miRNAs [33,34], and strong evidence has been provided for their role in cancer.

The loss of miR-15a and miR-16-1, due to chromosomal deletion of the locus 13q14 or germline mutation in their primary precursor, was associated with the development of the indolent form of CLL [31]. Both miRNAs were found to regulate posttranscriptionally the expression of Bcl-2, an anti-apoptotic protein that is widely overexpressed in CLL [39], which supports the role of these miRNAs as tumor suppressors in CLL. Loss of miR-15a and miR-16-1 has also been observed in prostate cancer and multiple myeloma [40,41]. Similarly, members of the let-7 family of miRNAs were reported to map in genomic regions which are deleted in different human malignancies [32], and their downregulation is commonly observed in lung, breast and colon cancer [37,42,43]. The role of let-7 as tumor suppressor was clearly demonstrated in lung cancer, following the observation that downregulation of let-7 in lung tissues led to the constitutive overexpression of Ras and high-mobility group AT-hook 2 (HMGA2), both being oncoproteins that contribute to the pathogenesis of cancer [44,45,46]. Reduced let-7 expression was also shown to enhance c-Myc signaling in Burkitt lymphoma (BL) cells [47].

In addition to miR-15a/miR-16-1 and let-7, miR-29 family members (miR-29a, b, c) were shown to function as tumor suppressor miRNAs, their downregulation being associated with the development and progression of several human malignancies, including CLL, lung cancer, invasive breast cancer and hepatocellular carcinoma [36,37,43,48]. Interestingly, Fabbri and colleagues demonstrated that miR-29 can function as a tumor suppressor in lung cancer through interference with the methylation of tumor suppressor genes. By promoting the downregulation of the DNA methyltransferases 3A and B (DNMT3A and 3B), miR-29 induces re-expression of methylation-silenced tumor suppressor genes, such as the fragile histidine triad protein (FHIT) and the WW domain containing oxidoreductase (WWOX) [49].

The miR-34 family, which comprises miR-34a, b and c, has also received considerable attention for its potential role as tumor suppressor in several human malignancies. Decreased expression of miR-34 has been observed in lung, ovarian, CLL and colorectal cancer [50,51,52,53]. Reduced levels of miR-34a were also observed in human gliomas, which correlated with increased expression of the target oncogenes c-Met, Notch-1/2 and cyclin-dependent kinase 6 (CDK6) in glioma and stem cells [54]. Interestingly, several reports indicate that miR-34 family members are direct transcriptional targets of the tumor suppressor p53 and suggest that some cellular roles of p53, including those related to the regulation of cellular proliferation and apoptosis, could be mediated by these miRNAs [55,56].

### 3.2. MiRNAs as Oncogenes

MiRNAs act as oncogenes when their increased expression or gain of function contributes to the development of a malignant cell phenotype [57] (Figure 1). One of the best-characterized oncogenic miRNAs is miR-17-92, a polycistronic cluster comprising six miRNAs (miR-17, miR-18a, miR-19a, miR-20a, miR-19b-1, and miR-92-1), that maps at 13q31.3, a region amplified in several types of lymphoma and solid tumors [58,59]. Expression profiling studies revealed widespread overexpression of these miRNAs in a large number of human hematopoietic malignancies and solid tumors, including breast, colon, lung, pancreatic, prostate and stomach cancers [35,60]. The members of the miR-17-92 cluster were shown to promote tumor proliferation and induce angiogenesis through the activation of c-Myc [58,60,61], which is frequently activated in cancer. Interestingly, O’Donnell and colleagues also reported that the transcription of the miR-17-92 cluster is directly transactivated by c-Myc [62], which suggests the existence of a feedback mechanism to enhance pro-oncogenic signaling. The miR-17-92 cluster was also shown to enhance proliferation by activating several members of the E2F family of transcription factors—E2F1, E2F2, E2F3—which induce the expression of genes that drive cell cycle progression from G1 into S phase, and by inhibiting the cyclin-dependent kinase inhibitor 1A (CDKN1A, also known as p21), a potent negative regulator of the G1-S checkpoint [63].

Strong evidence also suggests that miR-21 functions as an oncogene. Overexpression of this miRNA has been observed in numerous human malignancies, including colon, stomach, pancreas, prostate, lung, breast and liver cancer [35,43,64,65], being associated to important cancer hallmarks, such as uncontrolled cell proliferation, decreased apoptosis, invasion and migration [66]. Several studies also demonstrated that miR-21 dysregulation contributes to the pathogenesis of glioblastoma (GBM) [67,68,69]. MiR-21 promotes tumor cell proliferation by inhibiting PDCD4 (programmed cell death protein 4), a tumor suppressor that prevents cell cycle progression via activation of the cyclin-dependent kinase 1 (Cdk1) inhibitor p21 [70]. MiR-21 was also shown to target several components of p53, TGF-β and mitochondrial apoptotic networks, in order to decrease apoptotic activation in GBM cells [71], and to enhance tumor cell migration by inhibiting the matrix metalloproteinase (MMP) regulators RECK and TIMP3 [72].

Another miRNA with a clear role in the pathology of cancer is miR-10b. This miRNA is involved in the later stages of malignancy, by promoting the invasion of cancer cells into the surrounding stroma and metastasis to distant sites [73,74]. MiR-10b was found to be overexpressed in metastic samples of breast and hepatocellular carcinomas, when compared with tumor samples from metastasis-free patients [75,76], and patient samples from pancreatic adenocarcinomas [64] and glioblastomas [77], two types of extremely invasive/metastic cancer. It was proposed that miR-10b promotes invasion and metastasis by suppressing the translation of homeobox D10 (HOXD10), a transcriptional repressor known to inhibit the expression of several pro-metastatic genes, including the Ras homolog gene family member C (RHOC) [74]. In addition to translation suppression of HOXD10, an E-cadherin-related mechanism has been proposed to explain the role of miR-10b in breast cancer metastasis [78].

MiRNA function can, nevertheless, vary according to the tissue and its transcriptome, including the miRNA targets expressed in that particular tissue [3]. In addition to its well known oncogenic role, miR-17-5p, a member of the miR-17-92 cluster, was reported to be downregulated in breast cancer cells [79]. Interestingly, decreased cellular proliferation was observed following the overexpression of miR-17-5p in cultured breast cancer cells, thus suggesting that this miRNA can act as a tumor suppressor in breast cancer [79]. Similarly, the inhibition of miR-21 (a miRNA overexpressed in a wide variety of human tumors) was associated with increased cell growth in cervical cancer cells [80]. Therefore, as stated by Croce and collaborators, miRNAs should not be classified as oncogenes or tumor suppressors unless the tissue or cell type involved in their action is specified [3].

Although the examples presented above constitute a small subset of miRNAs implicated in cancer development, they emphasize that targeting aberrantly expressed miRNAs has potential to impact future cancer therapies. Strategies for manipulating the expression of miRNAs will be discussed below.

## 4. Therapeutic Modulation of MiRNAs

Two major challenges are associated with the manipulation of miRNA function. The first concerns the identification of molecules that can effectively inhibit or “mimic” mature miRNAs, in order to achieve losses or gains of miRNA function, respectively. The second challenge concerns the efficient delivery of these molecules to the specific targeted sites.

### 4.1. Silencing of MiRNAs: Targeting MiRNAs Overexpressed in Cancer

The remarkable capacity of single-stranded or double-stranded DNA or RNA analogs to inhibit the activity of selected single-stranded genetic sequences has been explored in therapeutic approaches for several human gene-related diseases, including cancer. As mature miRNAs are short oligonucleotides, their inhibition can be achieved by base-pairing with complementary oligonucleotide sequences.

Multiple steps in the miRNA biogenesis pathway can be targeted with antisense oligonucleotides (ASOs) to repress miRNA production or function [81]. In this regard, targeting the loop structure of the pre-miRNA was reported by Lee and colleagues as an interesting approach [82], although this was shown not to be very effective, possibly due to the difficulty in accessing the loop region. Inhibition of Drosha and Dicer processing of pri-miRNAs and pre-miRNAs, respectively, was achieved with morpholino ASOs in zebrafish [83]. Although effective, this approach may have limited application in mammalian systems due to the slow turnover of the majority of mature mammalian miRNAs [84,85], which restrains the timing of inhibition of miRNA activity.

Currently, ASOs complementary to the mature miRNA (also known as anti-miRNA oligonucleotides, AMOs), and designed to block its function in the miRNP silencing complex, constitute the most effective technology for controlling miRNA expression, experimentally and/or therapeutically [86]. In this regard, the addition of chemical groups to the 2'-hydroxyl group (at the C2 carbon of the ribose) was shown to be particularly effective in increasing the binding affinity for RNA and protecting the AMOs from nuclease degradation. Studies with AMOs containing methylated hydroxyl groups (2'-OMe) revealed increased resistance to nuclease cleavage and improved binding affinity to RNA compared to unmodified sequences [15,87]. When conjugated with a phosphorothioate backbone, intravenously-administered 2'-OMe-AMOs were also effective in inhibiting miRNA function in different animal tissues [88]. The addition of methoxyethyl (2'-MOE) or fluorine (2'-F) groups further enhanced the activity of AMOs against the target miRNA, when compared to the simpler 2'-O-methyl modification [89]. The strongest affinity for the target miRNA was, nevertheless, obtained with locked nucleic acid (LNA)-modified AMOs, which contain a methylene linker bridging the 2'-O-oxygen to the 4'-position that confers increased thermodynamic stability [90,91]. Although 2' modifications were shown to improve affinity to target RNA, their anti-miRNA activity was not fully correlated with affinity [90], suggesting that other variables may also be important for effective miRNA inhibition.

Synthetic polymers similar to RNA and DNA, designated peptide nucleic acids (PNAs), have also been described as excellent candidates for antisense therapies [92]. As opposed to the ribose and deoxyribose sugar backbone, PNAs contain a polyamide backbone composed of repeating *N*-(2-aminoethyl)-glycine units linked by peptide bonds [92]. Since the backbone of PNA is neutrally charged (contains no charged phosphate groups), the binding between PNA and DNA or RNA strands is stronger than that between strands of DNA and/or RNA, where the electrostatic repulsions contribute to decrease the duplex stability [93]. PNAs are not easily recognized by either nucleases or proteases, making them resistant to enzyme degradation, and can be easily modified to increase miRNA targeting [94]. Indeed, unmodified PNAs cannot readily cross cell membranes to enter the cytosol and, therefore, PNAs are usually coupled to targeting molecules, such as CPPs, to improve cytosolic delivery [95]. Efficient PNA-mediated miRNA inhibition was already reported in *in vitro* [94] and *in vivo* studies [96]. In addition to the normal AMOs, which contain only one binding site for the target miRNA, a different class of miRNA inhibitors containing multiple binding sites per molecule-designated miRNA sponges—have been developed.

The concept of miRNA sponge was introduced by Ebert and coworkers [97]. The authors reasoned that an mRNA-like transcript containing multiple complementary binding sites for an endogenous miRNA could bind the miRNA and block its activity. To achieve high levels of expression, they constructed plasmids encoding tandemly arrayed miRNA binding sites (MBS), driven by the CMV promoter. Aiming to prevent cleavage of the transcript containing the MBS, the authors introduced central mismatches in the miRNA/transcript duplex at positions 9–12. Upon transient transfection into mammalian cells and transcription by the RNA polymerase II, the transcripts (miRNA sponges) were at least as effective as LNA-modified AMOs in inhibiting not only one miRNA but also multiple members of a miRNA family [97]. Based on the work of Ebert, Kluiver and colleagues developed a fast and versatile method to generate stably-expressed miRNA sponges containing 10 or more miRNA binding sites [98]. Moreover, the authors reported that constructions containing multiple binding sites for two different miRNAs were efficient in inhibiting simultaneously both target miRNAs.

Since overexpression of miRNAs has been associated with several steps of the tumorigenic process, modulating their levels could provide therapeutic benefit. Indeed, encouraging results from *in vitro* and *in vivo* studies using AMO-based strategies have already been achieved.

Due to its considerable overexpression in a wide range of human tumors, miR-21 has been targeted in several anti-cancer AMO-based strategies. Knockdown of miR-21 in cultured hepatocellular cancer cells resulted in increased apoptosis and suppressed cell growth [64], while AMO-mediated miR-21 inhibition in androgen-independent prostate cancer cell lines (DU145 and PC-3) increased cell sensitivity to apoptosis and inhibited cell motility and invasion, without affecting tumor cell proliferation [99]. Similarly, transfection of breast cancer cells with anti-miR-21 oligonucleotides suppressed both cell proliferation *in vitro* and tumor growth in a xenograft mouse model [100]. Seike and colleagues reported that EGFR mutations are generally associated with increased miR-21 expression in nonsmoking lung cancer patients, and AMO-mediated miR-21 knockdown sensitized cancer cells to the EGFR-tyrosine kinase inhibitor AG1478 [101]. In a separate study, transgenic manipulation of miR-21 and the targeted delivery of anti-miR-21 oligonucleotides were shown to slow down tumor progression in a Ras-driven murine model of lung cancer [102].

Two different studies in cultured pancreatic cancer cells demonstrated that oligonucleotide-mediated miR-221 silencing results in increased apoptotic activity, decreased tumor cell proliferation [103] and increased cytotoxicity of the anti-cancer agent benzyl isothiocyanate [104]. Furthermore, 2'-O-Me phosphorothioate-modified anti-miR-221 oligonucleotides were shown to decrease proliferation of cultured hepatocellular cancer cells [105]. When tested in a mouse model of disease, the administration of a cholesterol-modified isoform of anti-miR-221 not only improved pharmacokinetics and liver tissue distribution, compared to unmodified oligonucleotide, but also reduced miR-221 levels in the liver (within a week of intravenous administration), produced significant antitumor activity and increased animal survival [105]. Similarly, the therapeutic silencing of miR-10b with cholesterol-modified AMOs (antagomirs) suppressed metastasis in a mouse mammary tumor model [106].

MiRNA sponges were also shown to be effective in inhibiting miRNA activity. Kluiver and colleagues demonstrated that the combined inhibition of miRNAs of the miR-17-92 cluster, using sponges containing binding sites for several elements of this family, was significantly more effective in inhibiting the proliferation of cultured B-cell lymphoma cells, than individual miRNAs [98].

### 4.2. Overexpression of MiRNAs: Re-Expressing MiRNAs Downregulated in Cancer

Two different strategies, involving miRNA mimics and plasmid or virally-encoded miRNA constructs, have been widely used to restore miRNA expression in cells, aiming at a therapeutic benefit.

MiRNA mimics are double-stranded RNA molecules similar to the endogenous Dicer product (miRNA:miRNA* duplex), composed of a guide strand identical to the mature miRNA and a passenger strand that is partially or fully complementary to the guide strand [107]. Due to their unfavorable physicochemical characteristics for *in vivo* administration, miRNA mimics can be chemically modified to increase protection from nuclease degradation, decrease innate immune system activation, reduce the incidence of off-target effects and improve pharmacodynamics [108]. In this regard, the addition of methyl (2'-O-Me), methoxyethyl (2'-MOE) or fluorine (2'-F) groups (or their combination) to the ribose ring was shown to enhance the stability of miRNA mimics [109,110]. Aromatic compounds, such as 3'-benzene-pyridine, were able to increase protection from nuclease degradation and enhance activity when added to the 3' end of miRNA mimics [111]. The guided strand can also be modified to enhance the miRNA activity in the miRNP complex. Indeed, the addition of 2'-OMe modifications at the 3' end and the presence of a 2-nucleotide (nt) 3' overhang assist with miRNP loading and degradation of the passenger strand [107,112].

Recently, a new class of miRNA mimics has been described. As opposed to the traditional double-stranded miRNA mimics, Chorn and colleagues demonstrated that modified single-stranded miRNA mimics also exhibit significant argonaute-mediated miRNA seed-based activity in cultured cells [113]. However, the potency of single-stranded mimics was inferior to that achieved with double-stranded oligonucleotides, which suggested that additional modifications should be explored, aiming at improving the efficacy of these molecules [113].

An alternative strategy for therapeutic miRNA replacement involves the expression of a shRNA or pri-miRNA mimic from a plasmid or viral construct. When compared to the delivery of double-stranded miRNA mimics, this approach provides a more stable expression of the mature miRNA and allows the expression of multiple miRNAs from one transcript [114].

ShRNAs are structurally similar to pre-miRNAs, with a base-paired stem, a small loop and a 3'-end UU overhang [115]. Expression of shRNAs is usually driven by RNA polymerase III promoters, including the H1 and U6, as they are involved in the production of small cellular transcripts and use precise initiation and termination sites [115,116]. However, both promote high levels of shRNA expression, which can elicit toxicity [117]. Indeed, overexpression of shRNAs was already shown to saturate both exportin-5 and Ago2, which triggers a global dysfunction of the endogenous miRNA pathway [115,118]. As opposed to shRNAs, pri-miRNA mimics are usually transcribed from low expression RNA polymerase II promoters, which can drive tissue-specific expression [117], and further processed by Drosha, thus reducing the risk of saturation of the endogenous miRNA pathway. In this regard, reduced *in vivo* toxicity was reported following miRNA re-expression via pri-miRNA mimics, when compared to that observed following miRNA re-expression via shRNAs [119,120]. Pri-miRNA mimics were, nevertheless, suggested to be less efficient in promoting the silencing of target mRNAs, when compared to matched shRNAs [119,120].

A common hurdle to both shRNAs and pri-miRNA mimics is the existence of off-target effects associated with their application. As stated by Sybley and collaborators, complementary base pairing with as little as 8-nt homology is sufficient to cause translational repression of nontarget genes when multiple target matches are present within their 3' UTRs [117,121]. Therefore, careful design of shRNA or pri-miRNA mimics constructs should be considered in order to reduce the off-target effects associated with these technologies.

Encouraging results from *in vitro* and *in vivo* studies using miRNA-expressing strategies have also reported. Enforced expression of miR-34a in human cultured p53-mutant prostate cancer (PCa) cells induced cell-cycle arrest, apoptosis or senescence [122] and, most importantly, re-expression of this miRNA in CD44^+^ PCa cells blocked tumor progression and metastasis following orthotopic tumor cell implantation in immunocompromised mice [122]. Similarly, re-expression of miR-34a in human colon cancer cells (HCT116 and RKO) caused a significant inhibition of cell proliferation and induced senescence-like phenotypes in these cell lines [123]. Importantly, intratumoral administration of complexes prepared by association of miR-34a and atelocollagen suppressed tumor growth in subcutaneous mice xenograft tumors [123]. In a different study, reduced tumor cell proliferation, migration and invasion were observed in cultured breast cancer cells following exogenous expression of miR-34a [124].

The transient overexpression of let-7a and let-7f, two dominant isoforms of the let-7 family in A549 lung adenocarcinoma cells, suppressed tumor cell proliferation [125]. Similarly, overexpression of let-7 in these cells and HepG2 liver cancer cells repressed cell cycle progression and cell division [126]. Let-7 overexpression was also reported to induce apoptosis and cell cycle arrest in colon cancer and Burkitt's lymphoma (BL) cell lines [41,46].

**Figure 2:**
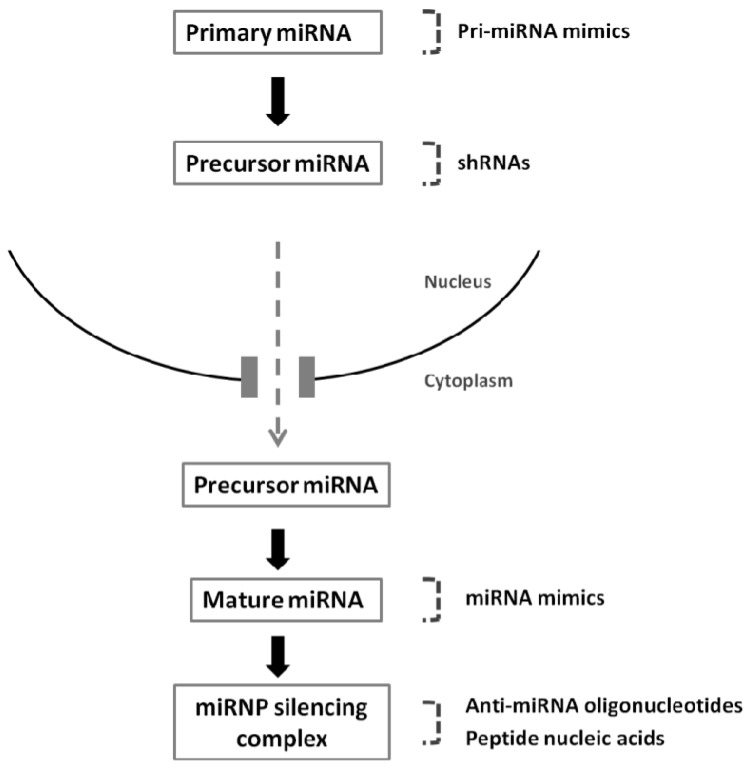
Strategies that are mostly employed to modulate miRNA expression. Constructs used to re-express or silence mature miRNAs: virally-mediated nuclear expression of pri-miRNA mimics or shRNAs, cytoplasmic delivery of double-stranded miRNA mimics, anti-miRNA oligonucleotides and peptide nucleic acids.

Decreased expression of members of the miR-29 family (miR-29a, b, c) was observed in various malignancies that contain aberrant DNA hypermethylation patterns, including lung cancer [48,127]. In this regard, the enforced expression of miR-29 in lung cancer cell lines led to reduced global DNA methylation, induced re-expression of methylation-silenced tumor suppressor genes, such as FHIT and WWOX, and inhibited tumor cell proliferation *in vitro* and tumor growth *in vivo* [48]. In CLL, the loss of miR-15a and miR-16-1 was associated with decreased apoptotic activity due to the overexpression of the anti-apoptotic protein Bcl-2, while miR-15a/miR-16-1 reconstitution increased apoptosis through repression of Bcl-2 mRNA translation [38]. Furthermore, overexpression of miR-7, a miRNA involved in the repression of the pro-oncogenic Akt pathway, reduced the proliferation and invasion in different GBM cell lines and in one GBM stem cell line [128], while expression of miR-128 inhibited the proliferation of glioma cells by decreasing the levels of E2F3a [129]. Figure 2 depicts the different strategies developed for the therapeutic modulation of miRNA expression.

Although modified AMOs and miRNA mimics have been successfully tested for miRNA modulation in liver diseases [130,131,132], including hepatocellular cancer [105,133], by taking advantage of the physiological role of the liver as the major blood filtration organ. Nevertheless, their widespread application to other non-hepatic diseases is restricted by the lack of tissue specificity. Being negatively charged molecules, miRNA inhibitors and miRNA mimics do not readily bind to cell plasma membrane and, with very few exceptions [134], do not cross the blood-brain barrier [135,136], which limits their use to the treatment of brain malignancies. Therefore, the successful *in vivo* nucleic acid delivery requires the development of carriers that can increase bioavailability, protect the nucleic acids from nuclease degradation and enhance their uptake by the target cells, while sparing the normal tissues.

### 4.3. Delivery of Nucleic Acids to Modulate miRNA Function

The discovery of RNAi-mediated mechanisms of gene silencing and the development of suitable carriers for *in vitro* and *in vivo* delivery of siRNAs and shRNAs [118,137], paved the way for establishing technical approaches that can be applied in miRNA-based therapies. Viral and non-viral vectors, particularly lipid- and polymer-based carriers, have been successfully tested for siRNA/shRNA delivery in preclinical studies [138,139], and, more recently, employed for delivery of AMOs or miRNA-expressing constructs in both cellular and animal models.

#### 4.3.1. Viral Vectors

Due to their high transduction efficiency, viral vectors have been extensively used for delivering miRNA-expressing constructs to cancer cells in miRNA-based therapeutics (Table 1). The systemic administration of an AAV8 encoding miR-26a, which is highly downregulated in hepatocellular cancer and induces cell cycle arrest by targeting the cyclins D2 and E2, resulted in inhibition of cancer cell proliferation, induction of tumor-specific apoptosis and significant tumor regression, without toxicity [140]. Similarly, the intranasal administration of adenovirally-coded let-7 significantly reduced tumor burden in an orthotopic mouse model of NSCLC [141]. Lentiviral vectors were also successfully used for the reestablishment of miRNA expression in tumors. In a xenograft prostate cancer mice model, the intratumoral injection of lentivirally-coded miR-15-16 led to growth arrest within 1 week of treatment and considerable volume regression thereafter, whereas no significant alterations in tumor growth were observed in animals treated with an empty viral vector [142]. Similarly, the intratumoral administration of lentiviruses coding for a sequence targeting miR-21 was shown to enhance tumor cell death by apoptosis and, when combined with the anti-neoplastic drug gentabicine, to induce tumor regression [143].

**Table 1:** MiRNAs as targets for cancer therapy through delivery of anti-miRNA oligonucleotides or miRNA-expressing constructs mediated by viral and non-viral vectors.

Carrier		Disease	Target miRNA (s) and role in cancer	References
**Viruses**	**Adeno-associated viruses**(AAV8)	Hepatocellular cancer	**miR-26** tumor suppressor	[140]
	**Adenoviruses**	Lung cancer	**let-7** tumor suppressor	[141]
	**Adenoviruses **	Glioblastoma	**miR-145** tumor suppressor	[144]
	**Adenoviruses**	Glioblastoma	**miR-221-222** oncogene	[145]
	**Lentiviruses**	Prostate cancer	**miR-15-16** tumor suppressor	[142]
	**Lentiviruses**	Pancreatic cancer	**miR-21** oncogene	[143]
**Lipid-based nanoparticles**	**Cationic liposomes**	Breast cancer	**miR-34a** tumor suppressor	[124]
	**Cationic liposomes**	Pancreatic cancer	**miR-34a, miR-143-145** tumor suppressors	[146]
	**Neutral lipid emulsion** **©**	Lung cancer	**miR-34a, let-7** tumor suppressors	[147]
	**Stable nucleic acid lipid particles**	Glioblastoma	**miR-21** oncogene	[148]
**Polymer-based nanoparticles**	**Polyurethane**	Glioblastoma	**miR-145** tumor suppressor	[149]
	**Poly(lactic-co-glycolic acid)**	Lymphoma	**miR-155** oncogene	[96]
	**Polyamidoamine**	Glioblastoma	**miR-21** oncogene	[150]

Viral vectors have also been applied in miRNA-based therapeutic strategies towards GBM. Lee and colleagues took advantage of the abundant expression of the enzyme telomerase reverse transcriptase (hTERT) in cancer cells and developed a multimodal GBM-targeting approach, combining hTERT-targeting ribozyme-controlled HSV-tk expression with overexpression of miR-145, a miRNA that is usually downregulated in GBM [144]. For this purpose, the authors constructed adenoviral vectors that express hTERT.Rz.HSVtk and miR-145 under control of the CMV promoter, to ensure high expression of the transgene in the target cells. In a xenograft mice model, the intratumoral administration of the adenovirus harboring the HSV-tk expression cassette plus miR-145, combined with intraperitoneal injection of ganciclovir, resulted in increased animal survival, when compared to that observed with the administration of virus coding for HSV-tk or miR-145 *per se* [144]. Adenoviruses encoding shRNAs targeting mature miRNAs have also been developed for GBM therapy. Wang and colleagues constructed an adenoviral vector expressing shRNAs that co-repress the expression of miR-221 and miR-222 [145]. Upon transduction of cultured GBM cells, decreased levels of these miRNAs were detected, which were associated with increased expression of their target p27kip1, cell cycle arrest in G1 phase and increased apoptosis [145].

#### 4.3.2. Non-Viral Vectors

Synthetic non-viral vectors, including lipid- and polymer-based nanoparticles, have also been widely tested for the delivery of miRNA-based nucleic acids into tumor cells, as illustrated in Table 1.

**Figure 3:**
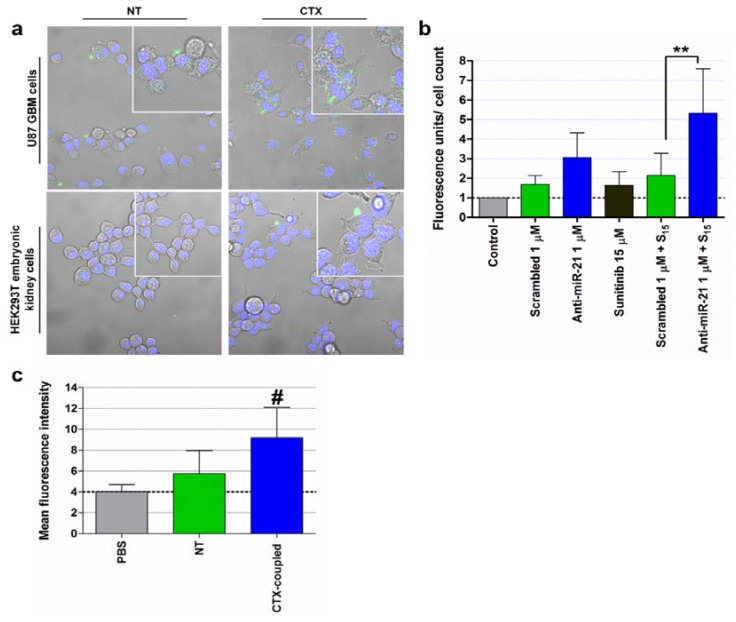
SNALP internalization in human U87 GBM and HEK293T embryonic kidney cells, caspase 3/7 activation, tumor cell proliferation and biodistribution of systemically-administered SNALP-formulated FAM-labeled oligonucleotides. (**a**) For evaluation of SNALP internalization, U87 and HEK293T cells were incubated with CTX-coupled (CTX) or nontargeted (NT) liposomes encapsulating FAM-labeled anti-miR-21 oligonucleotides (for 4 hours at 37°C), at a final oligonucleotide concentration of 1 μM. Cells were then rinsed twice with PBS, stained with DNA-specific Hoechst 33342 (blue) and then observed by confocal microscopy. (**b**) For evaluation of caspase 3/7 activation, U87 cells were incubated with CTX-coupled liposomes encapsulating anti-miR-21 or scrambled oligonucleotides for 4 hours, washed with PBS and further incubated for 24 hours with fresh medium. Cells were subsequently exposed to 15 μM of sunitinib for 24 hours, rinsed with PBS, after which caspase 3/7 activation was evaluated by the SensoLyte homogenous AMC caspase-3/7 assay (AnaSpec, San Jose, CA, USA). Results, presented as relative fluorescence units (RFU) with respect to control untreated cells, were normalized for the number of cells in each condition. Scrambled/anti-miR-21 1 μM + S_15_: cells transfected with scrambled or anti-miR-21 oligonucleotides and further incubated with 15 μM sunitinib. ^**^ *p* < 0.01 compared to cells incubated with SNALP-formulated scrambled oligonucleotides and further treated with 15 μM sunitinib. (**c**) Flow cytometry analysis (fluorescence intensity plots) of tumor homogenates from animals injected intravenously with CTX-coupled and NT liposomes encapsulating FAM-labeled siRNAs or saline solution (PBS). ^#^ *p* < 0.05 compared to animals injected with a similar amount of NT SNALP-formulated siRNAs. Results are presented as mean ± standard deviation of at least three different experiments.

Cationic liposome-mediated delivery of a plasmid encoding miR-34a (T-VISA-miR-34a) to cultured breast cancer cells was shown to result in the downregulation of several miR-34a target genes leading to significant suppression of breast cancer cell growth, migration and invasion [124]. Furthermore, intravenous injection of T-VISA-miR-34a: liposomal complex nanoparticles significantly inhibited tumor growth, prolonged survival, and did not induce systemic toxicity in an orthotopic mouse model of breast cancer [124]. Similarly, Pramanik and colleagues demonstrated that systemic intravenous delivery of liposome-formulated plasmid-encoded miR-34a or miR-143-145 inhibited tumor growth in subcutaneous and orthotopic pancreas MiaPaca-2 xenografts [146]. A different lipid-based formulation was also employed for the delivery of miR-34a and let-7 to a Kras-driven NSCLC mouse model. Trang and colleagues used a neutral lipid emulsion (NLE) that, when combined with synthetic miRNA mimics, forms nanoparticles in the nanometer diameter range and with a surface net charge close to zero [147]. In this regard, following intravenous administration of the lipid-based nanoparticles, a significant reduction in tumor growth was observed for animals treated with nanoparticles containg miR-34a or let-7, when compared to that observed for animals treated with nanoparticles containing a control miRNA mimic [147]. Importantly, a liposome-formulated miR-34a mimic is currently being tested in a phase I clinical trial in patients with unresectable primary liver cancer or metastatic cancer with liver involvement [151]. We have recently developed stable nucleic acid lipid particles (SNALPs), which were targeted towards GBM cells by covalent coupling of the peptide chlorotoxin (CTX) to the liposomal surface [148]. Our studies demonstrated that CTX-coupled SNALPs are able to efficiently and specifically deliver encapsulated anti-miR-21 oligonucleotides to cultured U87 GBM cells (Figure 3a), which resulted in a significant decrease in miR-21 expression, increased levels of the tumor suppressors PTEN and PDCD4, caspase activation (Figure 3b) and increased cytotoxicity of the tyrosine kinase inhibitor sunitinib. Importantly, *in vivo* studies revealed that the attachment of CTX to the liposomal surface enhances SNALP internalization into established intracranial tumors, when compared to that obtained for the non-targeted SNALPs (Figure 3c).

Delivery of miRNA-based nucleic acids, namely AMOs and miRNA mimics, to tumor cells has also been accomplished by using polymer-based nanoparticles. Some of the most commonly employed polymers include polyurethanes, poly(lactic-co-glycolic acid) (PLGA) and polyamidoamine (PAMAM) dendrimers (Table 1).

Polyurethanes are conventionally used in tissue engineering and gene delivery due to their biocompatibility and physicochemical properties [149,152]. When combined with PEI, cationic polyurethane (PU)-shortbranch PEI (PU-PEI) was shown to exhibit high transfection efficiency and low cytotoxicity *in vitro* and *in vivo* [149,153]. Using PU-PEI as a delivery vehicle, Yang and colleagues reported efficient delivery of miR-145 to CD133^+^ GBM cells, which resulted in a significant decrease in their tumorigenic potential and facilitated differentiation into CD133-negative cells [149]. Moreover, PU-PEI-mediated miR-145 expression in CD133^+^ cells suppressed the expression of anti-apoptotic and drug-resistance genes, while increasing the cell sensitivity to radiation and temozolomide (TMZ). When administered intratumorally in an orthotopic GBM-CD133^+^ xenograft mouse model, nanoparticle-formulated miR145 significantly reduced tumorigenesis and improved animal survival when combined with radiotherapy and TMZ, compared to that observed in animals treated with PU-PEI *per se* [149].

PLGA-based nanoparticles have also been extensively used to enhance the delivery of therapeutic agents to target cells, due to their biocompatibility and biodegradability [154]. In this regard, Babar and colleagues developed a delivery system by combining PLGA with PNAs targeting the oncogenic miR-155, and attached the CPP penetratin to the surface of the PLGA nanoparticles, in order to enhance cellular uptake [96]. Following incubation of cultured lymphoma cells with the generated nanoparticles (ANTP-NP), a significant increase in particle internalization, decreased expression of miR-155 and increased levels of the miR-155 target SHIP1 were achieved, when compared to that observed for cells incubated with nanoparticles lacking penetratin or nanoparticles loaded with a scrambled control. Moreover, upon intratumoral or systemic administration of ANTP-NP, a moderate (although significant) delay in tumor growth was detected in a subcutaneous xenograft mouse model of lymphoma [96].

Due to the presence of positively charged amino groups on their surface, PAMAM dendrimers can easily interact with nucleic acids to form complexes through charge-based interactions and protect them from nuclease degradation, while exhibiting minimal toxicity [150,155]. Moreover, the open nature of the dendritic architecture enables the entrapment of drugs within their core through electrostatic, hydrophobic and hydrogen bond interactions [156]. Ren and colleagues used PAMAM dendrimers as a carrier to co-deliver anti-miR-21 oligonucleotides and the drug 5-FU to human GBM cells and reported a significant increase of apoptosis and enhanced cytotoxicity of 5-FU, associated with a decrease in the invasive capacity of U251 GBM cells [150].

## 5. Conclusions

MiRNAs are a class of subtle gene regulators that, by targeting oncogenes and tumor suppressors, have the ability to modulate key cellular processes that define the cell phenotype. Abnormal expression of these small molecules was shown to play a causal role in different steps of tumorigenesis, from initiation and development to metastatic progression. Importantly, miRNA-based therapeutic strategies have been successfully applied in pre-clinical models for several human malignancies, thus emphasizing the enormous potential of miRNAs as therapeutic targets for neoplastic diseases. Future clinical trials should provide new insights into the safety and efficacy of the developed miRNA-based anti-cancer therapies.
